# Special Issue “Molecular Insight into Plant Bioactive Compounds”

**DOI:** 10.3390/ijms27052340

**Published:** 2026-03-02

**Authors:** Karolina Anna Wojtunik-Kulesza

**Affiliations:** Department of Inorganic Chemistry, Faculty of Pharmacy, Medical University of Lublin, W. Chodźki St 4a, 20-093 Lublin, Poland; karolina.wojtunik-kulesza@umlub.edu.pl

## 1. Introduction

Plants are an inexhaustible source of natural bioactive compounds. To date, thousands of secondary plant metabolites have been identified. In addition to their various roles in plants, including allelopathy and protection against abiotic stress or pathogens, the compounds have a wide range of biological activities that are significant for human health. Vegetables, fruits, and species rich in polyphenols, terpenoids, or alkaloids are characterized by diverse biological effects, including antioxidants, antibacterial and antiviral effects, metal ion reduction and chelation, and a positive impact on lifestyle-related diseases. An adequate amount of these extremely valuable compounds contributes to the prevention of many diseases, supports faster recovery, and helps maintain a youthful appearance [1,2].

The issue has deepened our understanding of secondary plant metabolites, including polyphenols, flavonoids, lignins, alkaloids, and terpenoids. The multidirectional scope of these published papers focuses on health-promoting properties, such as antioxidant properties, antimicrobial, enzyme-inhibitory, and procognitive activities, as well as providing a toxicity evaluation of these compounds. Various advanced techniques and methodologies, applied in both in vitro and in vivo studies, are also presented.

## 2. Overview of Published Articles

This Special Issue brings together a diverse collection of studies addressing the complex chemistry, biology, pharmacy, and therapeutic potential of plants as a source of bioactive compounds. This Special Issue accepted eight papers, including both original research articles and comprehensive reviews, highlighting key advances in the field. Collectively, they provide valuable molecular insights and highlight the importance of interdisciplinary collaboration among pharmacy, chemistry, and biology in the field of natural products research.

The research articles provide a detailed characterization of secondary plant metabolites, along with quality and quantity analyses, using advanced analytical methods. Granda-Santos et al. (contribution 1) presented a characterization of terpenoids in aromatic plants using Raman spectroscopy and GC-MS. The basis of the analyses was medicinal plants: *Tagetes filifolia*, *Aloysia citrodora*, *Cymbopogon citratus*, *Eucalyptus globulus*, *Chamaemelum nobile*, *Piper aduncum*, *Minthostachys mollis*, and *Rosmarinus officinalis*. The studies revealed differences in the proportion of oxygenated compounds compared with aromatic hydrocarbons and their influence on the differences between species; these were observed using Raman spectroscopy and GC-MS. Cis,cis-nepetalactone was identified in *M. mollis* for the first time. Comprehensive studies based on European *Phellinus* species were presented by Świderski et al. (contribution 2). The authors presented a multi-directional evaluation of the chemical potential and biological properties of samples collected from 22 *Phellinus* species. The most valuable biological activity was observed for *Phellinus igniarius*, *Fomitiporia robusta*, and *Porodaedalea pini*, which were the subject of detailed analyses. Interesting results are also presented by Valdivia-Padilla et al. (contribution 3), who focused on metabolomics’ characterization and bioinformatics studies of the bioactive compounds of *Psidium guajava* L. Studies based on principal component analysis (PCA) showed that *β*-caryophyllene was the predominant secondary plant metabolite in both genotypes of the tropical plant. Advanced analyses, including computational approaches, demonstrated significant interactions with target proteins, such as CB2, PPARα, BAX, BCL2, and AKT1. The results underscore the pharmacological promise of guava leaf metabolites in the context of natural product research and drug development. An evaluation of the pro-cognitive properties of citral was presented by Wojtunik-Kulesza et al. (contribution 4). A comprehensive evaluation based on in vitro, in vivo and ex vivo studies revealed the positive influence of citral (50 mg/kg; mice model) on the memory processes associated with acquisition. Additionally, GC-MS analysis revealed the presence of monoterpene in the blood and brain after the subchronic administration of citral and its accumulation in hippocampus. Li et al. (contribution 5) focused polyphenols from *Nymphaea candida* and their preventative activity against LPS-induced septic acute lung injury (ALI) in a mouse model. The polyphenols markedly alleviated lung damage, reduced the wet/dry ratio in the lung and reduced MPO activity. Additionally, the normalization of leukocyte profiles, lower level of pro-inflammatory cytokines, and suppression of TLR4/NF-κB and NLRP3 were observed. These findings indicate that NCTP exerts preventive effects against septic ALI, likely through the modulation of inflammatory signaling pathways.

The review articles collectively highlight the growing scientific interest in plant-derived bioactive compounds as complementary or alternative strategies for the prevention and treatment of major human diseases, particularly cancer, metabolic disorders, neurological conditions, and viral infections.

The review presented by Gökçen et al. (contribution 6) summarizes evidence that extracts from different parts of *Ficus carica* exhibit antiproliferative, pro-apoptotic and antioxidant effects in various in vitro cancer models. However, the findings vary depending on the plant part, extraction method and dosage, while in vivo and clinical data remain limited.

ElShamey et al. (contribution 7) explored the genetic basis of key bioactive compounds—GABA, resistant starch and alkaloids—in rice and barley, highlighting important genes and QTLs involved in their biosynthesis. These compounds contribute to cardiovascular health, glycemic control, gut microbiota modulation, and neuroprotection.

Bukowska (contribution 8) decided to review the therapeutic applications of cannabinoids from *Cannabis sativa*, particularly THC and CBD, which interact with the endocannabinoid system and exhibit anti-inflammatory, anticancer, and antiviral properties. Approved formulations are already used for epilepsy and multiple sclerosis, while emerging studies suggest broader potential in oncology and other chronic diseases.

## 3. Conclusions

This Special Issue brings together original research and review articles highlighting the chemical diversity and biological potential of plant secondary metabolites. Research papers provide detailed qualitative and quantitative characterization using advanced analytical techniques such as Raman spectroscopy, GC-MS, metabolomics, and computational approaches. Several studies demonstrated novel compounds, species-specific metabolic profiles, and significant biological activities. The review articles complement these findings by summarizing the current knowledge on plant-derived bioactive compounds in cancer, metabolic, neurological, and viral diseases. Collectively, the contributions underscore the translational potential of natural products while highlighting the need for further in vivo and clinical validation.

## Data Availability

Data are available upon request.
